# The virulence of *Streptococcus mutans* and the ability to form biofilms

**DOI:** 10.1007/s10096-013-1993-7

**Published:** 2013-10-24

**Authors:** W. Krzyściak, A. Jurczak, D. Kościelniak, B. Bystrowska, A. Skalniak

**Affiliations:** 1 Department of Medical Diagnostics, Faculty of Pharmacy, Medical College, Jagiellonian University, UJCM 9 Medyczna St., 30-688 Krakow, Poland; 2 Department of Pediatric Dentistry, Institute of Dentistry, Medical College, Jagiellonian University, Krakow, Poland; 3 Department of Toxicity, Faculty of Pharmacy, Medical College, Jagiellonian University, Krakow, Poland; 4 Genetics Laboratory, Department of Endocrinology, Medical College, Jagiellonian University, Krakow, Poland

## Abstract

In some diseases, a very important role is played by the ability of bacteria to form multi-dimensional complex structure known as biofilm. The most common disease of the oral cavity, known as dental caries, is a top leader. *Streptococcus mutans*, one of the many etiological factors of dental caries, is a microorganism which is able to acquire new properties allowing for the expression of pathogenicity determinants determining its virulence in specific environmental conditions. Through the mechanism of adhesion to a solid surface, *S. mutans* is capable of colonizing the oral cavity and also of forming bacterial biofilm. Additional properties enabling *S. mutans* to colonize the oral cavity include the ability to survive in an acidic environment and specific interaction with other microorganisms colonizing this ecosystem. This review is an attempt to establish which characteristics associated with biofilm formation—virulence determinants of *S. mutans*—are responsible for the development of dental caries. In order to extend the knowledge of the nature of *Streptococcus* infections, an attempt to face the following problems will be made: Biofilm formation as a complex process of protein–bacterium interaction. To what extent do microorganisms of the cariogenic flora exemplified by *S. mutans* differ in virulence determinants “expression” from microorganisms of physiological flora? How does the environment of the oral cavity and its microorganisms affect the biofilm formation of dominant species? How do selected inhibitors affect the biofilm formation of cariogenic microorganisms?

## Introduction

In the 18th century, it was demonstrated that microorganisms live not only in a single-cell form, but are also capable of forming clusters suspended in a mucilaginous extracellular substance. The pathogenicity of certain microbial species such as *Streptococcus mutans*, *Staphylococcus epidermidis*, *Legionella pneumophila* or *Pseudomonas aeruginosa* is inseparably associated with their ability to form biofilms on solid surfaces, e.g., tissues, catheters or implants [1–4]. This feature allows microorganisms to form three-dimensional structures in which cells become more resistant to antibiotics and changing environmental conditions, among others, through changes occurring as a result of interbacterial interactions and the presence of an exopolysaccharide matrix protecting the entire structure [5, 6].

## Microorganism pathogenicity

Interactions observed between pathogen and host have been the subject of research and discussion for many years. The historical approach to the problem of microorganism pathogenicity postulated by, amongst others, Koch, puts the pathogen or host in the main position by this featured affiliation to one of them. The term ‘pathogenicity determinants’ is related to the features which determine a microorganism’s ability to cause disease, but which themselves are not required for its survival [7]. Henderson et al. defined pathogenicity determinants as pathogen components which cause damage in a host organism; this may include factors vitally important for the microorganisms [8]. These definitions do not, however, take into account the role of host susceptibility to infection, indicating only that pathogen properties are responsible for disease development. According to these definitions, only those microorganisms which cause diseases in healthy people are pathogens and not opportunistic or commensal microorganisms which are only able to infect the hosts with immune system disorders.

Casadevall and Pirofski [9] proposed a new definition for pathogenicity and pathogenicity determinants of the microorganisms taking into account the state of host immunological defense. A given microorganism pathogenicity is expressed as a range of damage which is caused by the microorganism itself and by the immune system as a response to a pathogen. The state of a host’s immunological defense is the main determinant for bacterial pathogens and it determines the infection course and cure [9].

The determination of microorganism pathogenicity determinants according to the classification proposed by Casadevall and Pirofski (six classes) poses some interpretation problems in class I, since it seems that the key role in these infections is played by a host’s condition. The infected human constitutes a complex ecosystem in which homeostasis in the field of the bacteria-immune system is observed in the physiological state. The development of bacteremia occurs in cases of disturbances in this system, usually as a result of immunity deficiency or, more rarely, as a result of an overexpression of the features determining bacteria pathogenicity. There are, however, some bacteria, e.g., *S. salivarius* or most bacteria colonizing the oral cavity, which live in the human environment but are rarely described as pathogens, even in those patients with an impaired immune system. This suggests that even pathogens of a low virulence have to possess a minimum set of features determining their pathogenicity, which would allow them to penetrate and proliferate in a host organism.

In the study by Kreikemeyer et al. [10] concerning biofilm structure, the determinants of *Streptococcus* pathogenicity are related to the discovery of long filamentous structures similar to the pilus observed on bacteria surfaces. These structures exhibit adhesive properties and may play a key role in adhering to host cells and tissues, as well as in biofilm formation by pathogens of the *Streptococcus* species. The study demonstrated that the described pilus in *S. pyogenes* are responsible for bacteria adhesion and the formation of microcolonies on host cell surfaces, and also for aggregation itself, especially when influenced by human saliva. Also, in the case of *S. agalactiae*, these structures are engaged in pathogen interaction with host cells, e.g., adherence and bypassing the epithelium barrier. They are also responsible for bacteria clustering in a biofilm-like structure. In the pathogenesis of the diseases caused by *S. pneumoniae*, as in the case of other *Streptococcus* species, these structures are engaged in adhesion and colonization processes; however, compared to other *Streptococcus* species, the frequency of pilus occurrence is lower (less than 30 %) [11].

There are studies suggesting that *S. mutans* isolates have a greater ability to form biofilm than the isolates of other *Streptococcus* species, which colonize the human oral cavity environment [12, 13]. The studies focused on *S. mutans* cells, which form biofilm and proved that they exhibit a different expression of some proteins in comparison to planktonic cultures, e.g., an increase in exopolyphosphatase expression and a decrease of lactate dehydrogenase or pyruvate kinase expression [14]. Increased virulence of cells forming biofilm can also be associated with a higher tolerance to low pH, as compared to planktonic cultures.

The main components of the biofilm formed on the surface of teeth include: glucan (10–20 % of dry weight), fructan (1–2 % of dry weight) and proteins (40 % dry matter). Moreover, it differs from the surrounding saliva in terms of the levels of lipids, calcium, magnesium, fluorine, and phosphorus. In situ, in 80 % it consists of water [6]. Thus, an amorphous membrane formed in such conditions provides ideal conditions for bacterial survival and determines the virulence of the biofilm structure. Physical and biochemical matrix features allow for the adhesion of microorganisms, promote cohesion (the aggregation of cells), and act as a source of energy reservoirs. Moreover, limited diffusion to and from the biofilm helps to focus the ions and other nutrients in the microenvironment, such as biofilm; however, it hinders the penetration of substances from outside, including antibiotics [6]. It can be observed that bacteria in the biofilm resemble other organisms for which the clustering was also an evolutionary adaptation for survival.

The ability of bacteria of the *S. mutans* species to form biofilms is significant from a clinical point of view, mainly in the context of carries etiology; however, there are also single casuistic cases of infective endocarditis (IE) with the involvement of this bacteria [15, 16]. The development of IE is observed when endocardial damage occurs followed by the formation of a very small blood clot, in which platelets play a crucial role. If, at the same time, microorganisms enter the bloodstream, they may use these favorable growth conditions for deposition and biofilm formation.

Such microclots are formed at the interface of the mitral and tricuspid valves from the side of atria and from the side of chambers at the aortic and pulmonary valves [17]. The survival of *S. mutans* in the bloodstream, where it is very rarely observed after dental surgery, is associated with the presence of several virulence factors on the surface of bacteria, and these have been described in different cases of IE caused by this microorganism. First are those factors responsible for the increased resistance of proteins to phagocytosis, namely, the fibronectin-binding protein (so-called autolysin A, AtlA) [18] and serotype-specific polysaccharide [19]. Next, C antigens increased platelet aggregation, initiating the coagulation cascade process and, consequently, often leading to hypercoagulable states [20]. An equally important role is attributed to the collagen-binding protein (Cnm) [21], so that the *S. mutans* bacterium can adhere to the heart tissue and penetrate the endothelium of the coronary arteries, consequently leading to endocarditis [22].

## Biofilm formation as a complex process of protein–bacterium interaction

The most common oral cavity infectious disease in which an important causative role is played by biofilms formed by microorganisms on the teeth and gums surface is dental caries. One of the main etiological factors of the above-mentioned disease is *S. mutans* [23–25].

Caries is the most common childhood illness. It is estimated to occur five times more often than the second most common childhood illness, asthma [26]. The Decayed, Missing and Filled Teeth (DMFT) index rate was observed for Bolivia, Guatemala, Bosnia and Herzegovina, and Philippines. Directly behind them in contrast, the lowest DMFT index rate was observed for Russia, Europe’s Eastern Bloc including Poland, and most South American countries [27, 28].

Comparing the DMFT index rate for European countries, one can observe that the epidemiological situation in Poland is not ideal. In 2012, the DMFT index rate for 12-year-olds was estimated at 4.4, whereas the lowest observed in Holland was estimated at 0.9 and the highest in Latvia was estimated at 7.7. In terms of 6-year-olds, the situation deteriorates because, among the countries under investigation, the highest DMFT index rate equal to 5.1 was recorded in Poland. It is estimated that, in Poland, dental caries is observed in 35–50 % of children aged 2–3 years and in 50–60 % of children aged 3–4 years [27, 29]. According to alarming reports from the World Health Organization (WHO) and the National Institute of Public Health (PZH), dental caries is observed in almost 100 % of children aged 6–7 years. Caries in permanent teeth starts just after the dentition of the first molar teeth and has been reported in approximately 90 % of Polish 12-year-old children [30, 31].

In the view of the above data, it is extremely important to understand more fully the mechanisms of cariogenic strain activity on the basis of the biofilm formed by them, which may be applied in the prevention and early diagnostics of dental caries in children.

Biofilms present in the oral cavity are three-dimensional structures, consisting of bacterial strains anchored to solid surfaces such as tooth enamel, tooth roots or dental implants. They are embedded in an exopolysaccharide matrix [32]. Over 700 different bacterial species incorporating into biofilms have been identified so far [33, 34]. The structure and composition of the exopolysaccharide matrix is determined by the conditions existing in the oral cavity and change over time. The extracellular polysaccharides (EPS) also affect the physical and biochemical properties of the biofilm [6, 35].

**Fig. 1:**
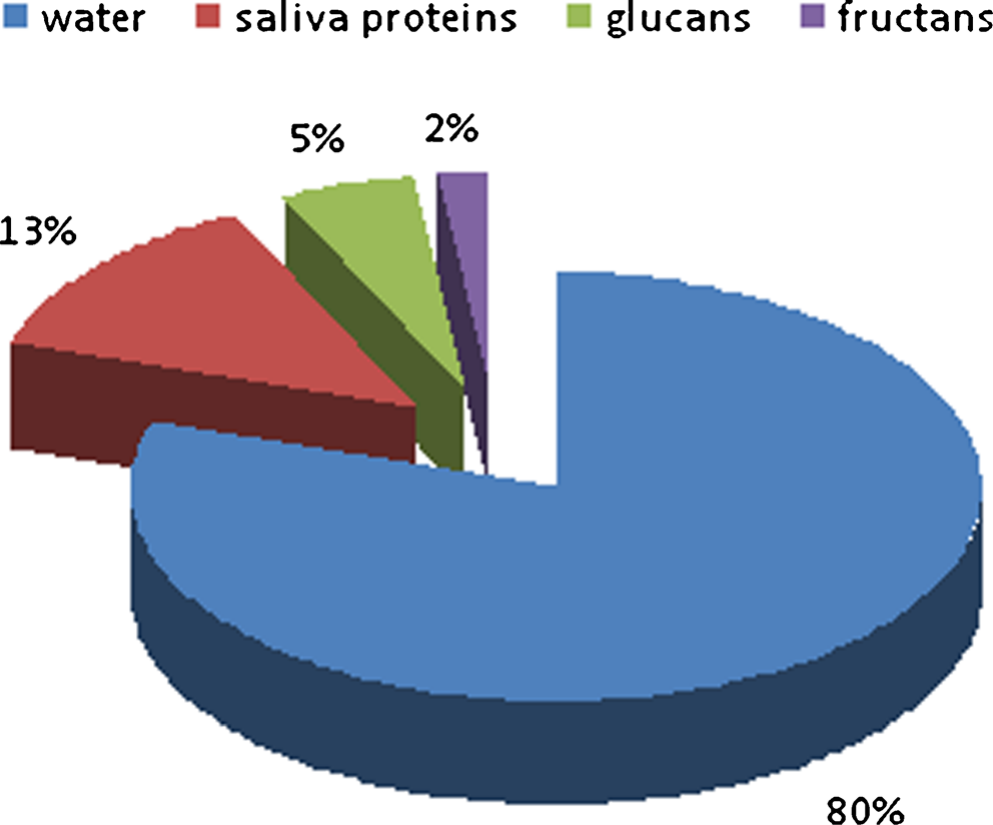
The percentage composition of polysaccharide matrix (the figure was prepared based on the data published by Bowen and Koo [6])

The primary sources of EPS are glucosyltransferase (GTF) and fructosyltransferase (Ftf), products of interaction with sucrose and starch hydrolysates. Exopolymers contained in the polysaccharide matrix form its stability and provide possibilities of binding bacterial cells [6, 35, 36]. Research on the composition of the polysaccharide matrix revealed the presence of components as shown in Fig. 1.

The process of biofilm formation begins with the coating of the tooth surface through the salivary pellicle [6, 32]. This pellicle is formed by salivary components (such as proline-rich proteins, amylase, lysozyme, histatin, peroxidase, mucin, and bacterial components, e.g., Ftf, Gtf, lipoteichoic acid) specifically adsorbed to the acquired enamel pellicle (AEP) [6, 37]. AEP is the basis for microorganism-induced biofilm formation colonizing the oral cavity [37]. Single *S. mutans* cells or their aggregates fuse with pellicles via two independent mechanisms: sucrose-dependent and sucrose-independent [23, 25, 32].

### The sucrose-dependent mechanism

#### Glucosyltransferases (GtfS)

The sucrose-dependent mechanism of plaque formation is based on glucosyltransferases (GTFB, -C, and-D) produced by *S. mutans* in combination with glucan-binding proteins (GBPs) [6, 25, 38]. Glucosyltransferases play critical roles in virulent dental plaque development and are responsible for glucans formation from sucrose. The synthesized glucans provide the possibility of both bacterial adhesion to the tooth enamel and microorganisms to each other. Thanks to this process, microcolonies are formed which favor the formation of biofilm. Each of the three types of Gtf plays a different, though similar role in biofilm formation and, therefore, the loss or mutation of one of them impairs the whole process [6, 38].

In vivo, GtfS very rapidly (approximately 1 min) adsorbs into the hydroxyapatite surface (sHA) of the enamel-coated salivary pellicle. The highest affinity for the sHA is reported for GTFC (formally known as GtfSI), which is a hydrophilic compound and produces a mixture of soluble (with mostly α-1,6-linkages) and insoluble glucans. However, it has a hydrophobic domain which is related to its affinity for dental plaque. This enables the interaction with saliva proteins in the pellicle, such as lysozyme or α-amylase. GtfB (known as GtfI), a glucosyltransferase, is primarily responsible for the interaction with other *S. mutans* bacteria which mainly synthesize insoluble glucan rich in α-1,3-linkages. It is responsible for the formation of highly differentiated microcolonies forming the structure of biofilm [39]. Its activity significantly increases when there is a glucose in the environment, which is not a typical situation and is observed extremely rarely [6, 40].

GtfD (GtfS) predominantly forms soluble, quickly metabolizable polysaccharides and acts as a primer for GtfB.

Glucosyltransferases also interact with the components of saliva: amylase, in consequence blocking the activity and adsorption to hydroxyapatite; lysozyme, which decreases the activity of GtfB, but does not affect the characteristics of glucans formed by the enzyme; peroxidase, which inhibits the activity of all three glycosyltransferases.

GtfS also have the ability to bind to other bacterial cells, even if these are not bacteria synthesizing their own glycosyltransferases. Therefore, cooperation between Gtfs and microorganisms allows for the building of a plate strongly attached to the teeth surface with stable bonds between bacterial cells [6, 35].

Genes encoding GtfB and GtfC lie close to each other, have a very similar amino acid composition (95 % homology), and are subject to the same regulatory processes. These genes are expressed in response to acidification of the environment or in situations of glucose or sucrose excess in the environment. Many factors additionally influence the physiological expression of these genes. One of them is catabolism and factors like RegM, an inhibitory regulator of *S. mutans* [6]. The protein regulating the catabolism in *Streptococcus* and *Staphylococcus* bacteria, RegM, is also known as catabolite control protein A (CCPA) [41]. Its regulatory function is based on the inhibition of genes involved in the utilization of alternative carbon sources and the activation of the expression of genes whose products are involved in the elimination of excess carbon from the cell [42–44]. Inactivation of the RegM of *S. mutans* results in a very strong decrease in the promoter expression of gtfBC [6]. Other factors are the products of genes: *luxS* (AI-2 autoinducer-coding synthesis), which affects the expression of *gtfB* and *gtfC* [6, 25, 45], and also *ropA* (encoding for the trigger factor) regulating the production of GtfB and GtfD [6, 23]. Also, the VicRK signal transduction system affects the physiological expression of Gtfs. In a mutant strain of *S. mutans* which does not have this system, a significant decrease in *gtfD* gene expression, as well as increased expression of the *gtfB* gene, was observed.

The *gtfD* gene lies distant from other genes, is characterized by lesser homology (50 %) and is subject to different regulatory mechanisms. Its expression is specifically induced by copper ions [6]. Apart from Gtfs, the synthesis and structure of glucans is also affected by enzymes such as mutanase and α-1,6-glucosidase. Therefore, the structure of glucans in the biofilm matrix is changeable, and in mature dental plaque, water-insoluble polysaccharides dominate [6, 46, 47].

#### Glucans binding of proteins (Gbps)

Another component of the sucrose-dependent mechanism is Gbps mediating the binding of bacteria to glucans. Four types of this protein are known: GbpA, -B, -C, and -D [6, 25, 38]. The GBPC protein (and probably GbpB) is associated with the bacterial cell wall and, therefore, acts as a specific receptor for glucan. All four types of proteins play a role in microorganism adhesion and biofilm formation; however, the GbpD protein seems to play a key role [6]. Research on the utility of GS5 (deletion of the *gbpB* gene) and UACA2 (a strain of the *gbpB* gene expression encoding antisense RNA) of *S. mutans* strains showed that the absence or mutation of the gene encoding GbpB results in a change of cell shape and a slowing down of its growth [38]. This disables the appropriate development of biofilm, which, instead of a diverse, dense formation, becomes a product of non-regular cell clusters surrounded by a matrix of unusual structure.

The expression patterns of GbpB which have been studied by Fujita et al. [40] may have some relationships with *S. mutans* virulence. Attention was paid to the relationship of the profiles and biological activity of the above-mentioned proteins with the virulence of isolated proteins. GbpB expression analysis isolates revealed the existence of several expression patterns. Strains that showed single and multiple bonding were classified as S and M strains, while strains without explicit GbpB expression were classified as N type. The GbpB expression pattern distribution was found to be different for the Japanese and Finnish clinical strains simultaneously.

For the Japanese strains, the highest frequency was reported for S (81 %), followed by the M (15 %) and N types (4 %), while in the Finnish population, the frequencies were estimated at 42 % for the M type, 37 % for the S type, and 21 % for the N type without the explicit expression of GbpB. Studies show a clear differentiation of each type depending on the origin of clinical isolates. Geographic variation seems to be crucial in determining the virulence of the strains, which, depending on the environment, do or do not acquire specific virulence factors, constituting a kind of adaptation which allows them to colonize a new ecological niche. An explanation of the distribution and specificity of GbpB expression patterns may be useful for the evaluation of *S. mutans* virulence in individual patients [40].

### The sucrose-independent mechanism

The sucrose-independent mechanism is not relevant in the virulence of *S. mutans*. In the second mechanism of adhesion (sucrose-independent), an interaction is observed between the adhesive particles of *S. mutans* and the AEP. Agglutinins found in saliva are involved in the process of adhesion and aggregation of *S. mutans* thanks to interaction with the I/II antigen, which is a multifunctional PI adhesin (also known as AgB, SpaP, or Pac1 adhesin) anchored in the bacterial cell wall, and encoded by the spaP gene [23–25, 48].

The protein family of Ag I/II, represented by SpaP, SspA, or SspB, is identified not only on the surface of *S. mutans*, but also on other microorganisms, such as *Streptococcus pyogenes*, *Streptococcus agalactiae*, or *Streptococcus suis* [48]. Genetic sequences encoding Ag I/II comprise six distinct regions. The most important of these are the A region rich in alanine and the P region rich in proline. Region V, located between them, is composed in the majority of different sequences found in individual strains. The A and V regions encode adhesive epitopes appearing on the surface of bacterial cells (so-called adhesive types) responsible for affinity to the salivary glycoproteins [49]. The contribution of the A, P, and V regions to the adhesion has been confirmed by studies using mutant strains [48]. None of them had the ability to adhere to solid surfaces coated with the salivary pellicle. The expression and biological activity of the P1 protein in *S. mutans* is also dependent on multiple gene products, i.e., luxS, ropA, and srtA genes (encoding the enzyme responsible for the attachment of P1 adhesin to the cell wall) [22, 50].

**Fig. 2:**
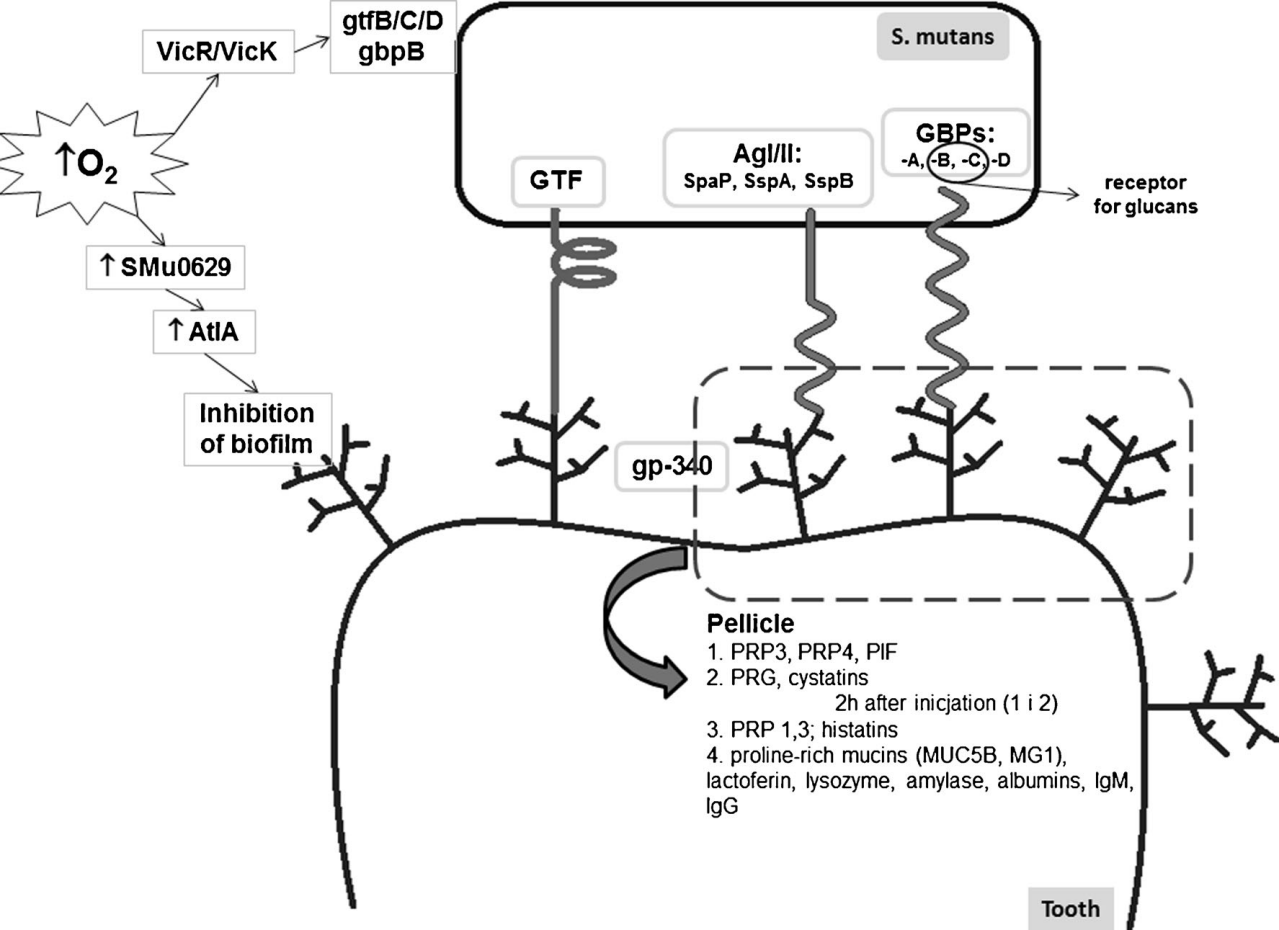
The function of *Streptococcus mutans* in the process of the formation of biofilms on the surface of teeth. Glycosyltransferases (GTF) are an indispensable element for the proper functioning of *S. mutans*. In the early phase of biofilm development, *S. mutans* are bound to the teeth surface. It is thought that this binding is the first step in the formation of plaque. Surface adhesins of *S. mutans* (so-called antigen I/II) interact with α-galactosides from saliva, forming the structure of pellicula. Other groups of compounds belonging to *S. mutans* and located on the surface of teeth which participate in the formation of pellicula are glucan-binding proteins (GBPs) or GTF. Salivary proteins with which can interact the surface adhesins of *S. mutans* can be divided into four groups depending on the time of pellicula formation. Proline-rich proteins (PRP) participate in the first stage of the formation of pellicula. Two hours later, enamel is formed by cystatins, peptides with high affinity to hydroxyapatite. Then, peptides with low molecular weight and with bacteriological properties against *S. mutans* start playing a role in this model. In the last stage, mucin-rich proteins, lactoferrin, lysozyme, amylase, albumin, or IgM and IgG antibodies are involved in the process. *S. mutans* are aggregated on the surface of teeth in the presence of saccharose. Additionally, GTF synthetizes extracellular glucans, which is another key step in the development of plaque. GBP is a *S. mutans* receptor that differs from GTF and specifically binds glucans. GTF contain a glucan-binding domain and, therefore, they act as receptors for glucans. Therefore, *S. mutans* binds initially developed glucan through GBP and GTF, which gives a basis for the aggregation of *S. mutans*

The SpaP protein and other proteins of the Ag I/II family specifically interact with glycoprotein-340 (gp-340) found in saliva. It is interesting that the gp-340 dissolved in the liquid phase of saliva plays a role in the aggregation of bacterial cells and, thus, purifies the oral cavity. However, if gp-340 is adsorbed on the surface of the teeth or gums, it acts as a receptor for surface bacterial adhesins initiating the adhesion process [22, 48] (Fig. 2). The Ag I/II protein family is also involved in the interaction between microorganisms, e.g., *Streptococcus gordonii* and *Porphyromonas gingivalis*, and in the aggregation of cells in the absence of gp-340 [48].

**Fig. 3:**
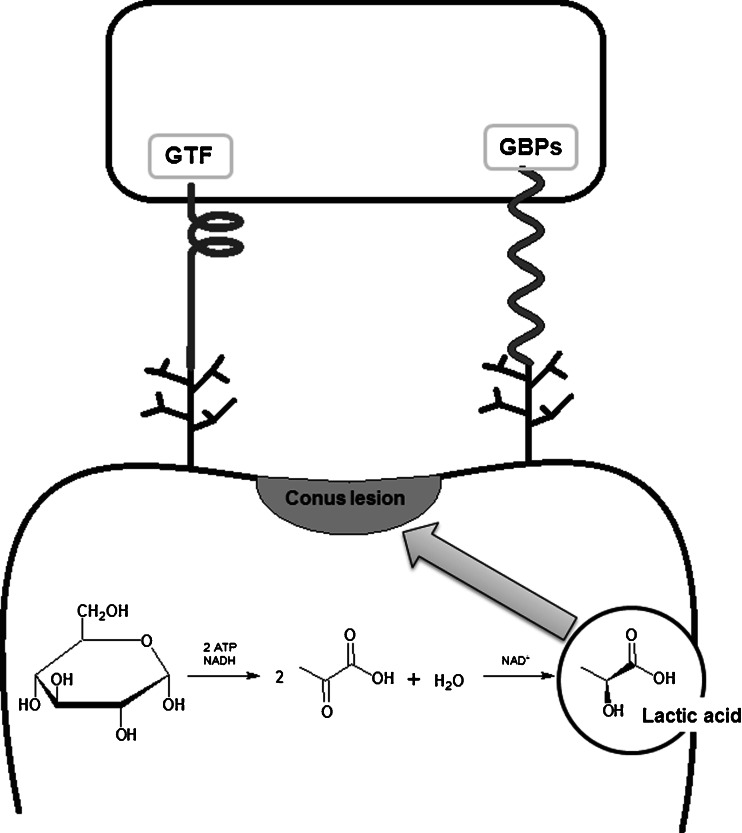
Production of lactic acid by *Streptococcus mutans*. Metabolism of various carbohydrates (including glucose and fructose) by bacterial biofilm. Production and secretion of a significant amount of lactic acid, which can cause demineralization of teeth structure that can finally result in the development of decay

A key role in the interactions between *S. mutans* and saliva agglutinins is played by the actual structure and location of P1 protein, as has been proven through the use of mutant strains (spaP and srtA) of this microorganism. Additionally, it has been shown that the lack of expression of the gene encoding sortase A enzyme (SrtA) results not only in an abnormal location of the P1 adhesin, but also of other surface proteins, which is not negligible in the context of bacterial aggregation ability [23]. The survival of *S. mutans* inside biofilms, at a very low pH inside microcolonies of other bacterial species (as observed in the oral cavity), should be considered as three mechanism-dependent phenomena which include: the upregulation system of F1F0-ATPase, biosynthesis of membrane fatty acids (for instance, FabM), and branched-chain amino acid (BCAA) (for instance, IIvC). This process may be the main driving force in the survival of *S. mutans* inside a mixed biofilm (Fig. 3); thus, innovative therapies aimed at inhibiting biofilm formation should be oriented along these three mechanisms and should be directed at all three simultaneously and not just in one of them [51].

### Salivary agglutinins

In the process of biofilm formation, a role equally important as that of surface bacterial adhesins is played by salivary agglutinins. This has been confirmed in vitro [23, 24]. Researchers have also used mutant bacterial strains of the major genes encoding the P1 protein to study the differences and interactions during biofilm formation. The results clearly indicate a less satisfactory process in the absence of salivary agglutinins, as well as significantly less sufficiency in the mutant strains compared to the parental strain [22, 23]. It has also been proven that the initial stage of adhesion and biofilm formation of *S. mutans* may be stimulated by salivary agglutinins and other salivary proteins (e.g., mucin of high molecular mass or acidic proline-rich proteins) (Fig. 2) [23, 52].

Under specific conditions, the expression of the virulence factors of *S. mutans* and biofilm formation in the oral cavity may be modulated in two ways: through the environment in which bacterial growth was reported as well as by the presence of other microorganisms, and the interactions between them [25].

Culturing *S. mutans* strains under aerobic conditions induces an 80 % reduction of bacterial ability to form biofilms [23]. Oxygen availability is the causative agent of variations in bacterial cell surface composition and modification of the production of autolysins and, specifically, in the signal transduction system of VicRK (Fig. 2). The production of AtlA autolysin is conditioned by the *SMu0629* gene expression of oxidoreductase activity. Under aerobic conditions, increased expression of this gene is being observed and, thus, the overproduction of AtlA autolysin, which inhibits the formation of biofilm. Vick kinase transducer is a system regulating the expression and activity of AtlA autolysin. In order to confirm oxygen-induced alterations in autolysin, studies on mutant strains for *SMu0629* and *vicK* genes were conducted. Strains from which the above-mentioned genes were removed adapted better to aerobic conditions and showed a greater ability to form biofilm as compared to the UA159 parental strain [52–54].

### Diet habits

Diet is another factor influencing the process of biofilm formation. The occurrence of dental caries is strongly correlated to diet. Meal cariogenicity is affected not only by the content of carbohydrates but also by the frequency of their consumption. Particular carbohydrates differ in their ability to cause caries. Saccharose is definitely the most cariogenic carbohydrate [55]. It constitutes the main center of *S. mutans* metabolism. These bacteria exhibit not only the ability to decompose this carbohydrate, but also produce glucans which are significant in interactions between tooth enamel and cariogenic bacteria [6].

The presence of high levels of carbohydrates in children’s diets, especially in the drinks given to children immediately before sleep or during the night, constitutes a significant factor in ECC development [56]. Falling asleep with a sweetened nipple or bottle in the mouth stimulates the formation of carious lesions. Saliva excretion decreases during sleep, which accelerates the cariogenic strength of the activity of acids derived from carbohydrate metabolism. The opinions concerning the effect of feeding with milk on dental caries development still remain controversial. According to researchers, cow milk does not contain high amounts of lactose and, moreover, no significant relationship with carious lesion formation has been demonstrated. Human milk, in turn, despite having higher amounts of lactose, also contains case in which adsorbs to the tooth surface and retards caries development. Moreover, it contains considerable amounts of calcium and phosphates, which additionally stimulate enamel remineralization [57]. Also, the significant influence of sparkling drinks and fruit juice on dental caries development in children and teenagers has been demonstrated. Acids common in sparkling drinks, such as citric or phosphoric acids, considerably lower the oral cavity pH and stimulate an exchange of calcium ions present in the enamel on hydrons. It has also been demonstrated that drinking through a straw elongates and increases liquid contact with the tooth surface, which unprofitably affects their structure [58].

It is not the level of carbohydrates in a meal but the frequency of consumption of meals with high carbohydrate levels that is a significant factor [59]. Also, time plays a substantial role in tooth mineralization. It has been demonstrated that alternate processes of enamel demineralization and remineralization occur during the day. The loss of hydroxyapatite mineral components is related to the period when bacteria fermenting carbohydrates may freely develop and synthesize acids into carbohydrates metabolism byproducts. Time acts in favor of cariogenic pathogens which adapt to low pH values, which favor the progress of the cariogenic process. The length of change in the period on the enamel surface is especially significant in cases of early childhood caries characterized by an aggressive course. Noticing the first changes on the upper incisor surface is extremely important, since carious lesions quickly spread on molar teeth [60]. Moreover, the changes in the daily rhythm of saliva excretion play a significant role in caries exposure. Saliva secretion is decreased during the night hours, which limits its ability to protect against enamel demineralization [58].

Currently, studies on the development of products to prevent dental caries development are being conducted. Some are aimed at inhibiting the development of biofilm through the influence on Gtfs. There are a number of synthetic and natural glycosyltransferase inhibitors, i.e., hop components, green tea, medicinal plant extracts, plant polyphenols of high molecular mass (e.g., curcumin), cardiolipin, putrescine, cadaverine, or hypochlorite compounds or metal ions (Fe^2+^, Zn^2+^, Cu^2+^). In order to increase their effectiveness, there is a tendency to develop formulations for oral hygiene which best use the potential of the above-mentioned substances [6, 61, 62].

## Biofilm structure

The formation of biofilm is a multistep and very complicated process. A number of relevant factors and conditions are required in the oral cavity for the process to run correctly.

Interactions occurring between agglutinins of saliva and bacteria, and simultaneously between microorganisms, might cause the formation of fur composed of cells beginning the colonization: *Actinomyces* species, *Streptococcus* species, *Lactobacillus* species, *Candida* species. They transform into different types of biofilm in the first layer of subgingival plaque. Biofilm maturation is followed by the aggregation of subsequent bacteria and their growth. After 7 days, the number of *Streptococcus* bacteria decreases, but the number of *Fusobacterium nucleatum* increases. After 3 weeks, intact subgingival plaque begins to resemble morphologically supragingival plaque [32].

In the architecture of supragingival biofilm, four layers can be distinguished. The first layer of biofilm consists of cells displaying little fluorescence relative to cells in the top layer. This indicates the presence of *Actinomyces* sp. In this layer, physiologically inactive or dead cells can also be found. In the intermediate layer, many spindle-shaped cells are found, whose fluorescence indicates *Fusobacterium nucleatum* and *Tannerella* sp., mostly *T. forsythia*. These bacteria can benefit from the proximal location of dead cells (e.g., they acquire sugar building their cell wall). The third layer of biofilm and the intermediate layer mainly consist of a bacterial cluster termed the *Cytophaga–Flavobacterium–Bacteroides* (CFB), which consists mainly of Gram-negative bacteria *Tannerella* sp., *Prevotella* sp., and *Bacteroidetes* sp.

Apart from this, long cigar-like *Synergistetes* bacteria from the A group forming palisade-like stroma are also found in this layer. These bacteria are in direct contact with the cells of the host immune system resembling polynuclear leukocytes, which, thereby, suggests their important role in the interactions occurring between host and biofilm. Observations of the top biofilm layer have shown that *Spirochaetes* bacteria are the most abundant. Among them, bacterial aggregates called rough and fine brushes are found [32].

More heterogeneous than subgingival biofilm is supragingival biofilm. It consists of two layers: basal and top, described in Table 1. All the above-mentioned factors and conditions provide an adequate oral microbial habitat for various kinds of microorganisms. The varied morphology of biofilm expands and targets attacks on potential anticaries compounds. At the same time, however, it is a complex structure, the variability of which makes it difficult to determine the destination points that underlie effective prevention related to the treatment of periodontal disease.

**Table 1:** Composition of supragingival biofilm (the table was prepared based on data published by Zijnge et al. [32])

Basal layer—attached to the surface of teeth and consists of four types of biofilm	The first type: - rod-shaped cells of *Actinomyces* located vertically towards the surface of the teeth
	The second type: - *Actinomyces* sp. - cocci chains, not identified as *Streptococcus*, located vertically towards the surface of the teeth
	The third type: - filamentous bacteria - *Streptococcus* creating distinct colonies around yeast cells (*Candida* sp.)
	The fourth type: - mainly *Streptococcus* developing in the vicinity of *Lactobacillus* sp. located vertically to the surface of the teeth
Top layer—covers all types of biofilm-forming basal layer	- *Streptococcus* sp. in a form of heterogeneous distributed cells, or as a flat, thin layer on the top - heterogeneous distributed bacteria creating CFB cluster - external layer: *Lactobacillus* sp. surrounded with cells with various morphology

## Interbacterial interactions and the process of biofilm formation

Interactions between microorganisms colonizing the oral cavity are consecutive major factors affecting the development of biofilm [25, 63, 64]. Interactions occurring between microorganisms can result in both the acceleration and inhibition of this process. Thus, the virulence of *S. mutans* depends not only on the environmental conditions of the oral cavity, but also on the composition of the bacterial flora.

The analysis of interactions between *S. mutans* (as one of the etiological factors of dental caries) and the components of physiological flora, e.g., *S. oralis*, *S. sanguinis*, or *Lactobacillus casei*, was made possible by the use of dual cultures [25]. In studies on this topic, single bacterial cultures acting as the control group were compared to dual cultures. The results of the experiments are summarized in Table 2.

**Table 2:** The results obtained in a study with the use of dual cultures (the table was prepared on the basis of data published by Wen et al. [25])

Dual culture	Biofilm formation	Expression of pathogenic factors of *S. mutans*			
		*spaP*	*gtfB*	*gbpB*	*luxS*
*S. mutans* + *S. oralis*	Not a relevant change	30-fold decrease	No decrease	30-fold decrease	15-fold decrease
*S. mutans* + *S. sanguinis*	Significantly important decrease	Small decrease	Small decrease	Small decrease	Change not statistically significant
*S. mutans* + *L. casei*	Small increase(around 2-fold)	40-fold decrease	40-fold decrease	40-fold decrease	7-fold decrease

The study of Zijnge at al. [32] suggests that *S. mutans* and *S. sanguinis* are microorganisms which possess an inhibitory effect on biofilm formation ability. Thus, taking into account this observation, researchers have decided to isolate and analyze proteins produced by *S. salivarius* [63]. Based on these studies, they found that substances responsible for biofilm formation inhibition are: fructosyltransferase (Ftf) and exo-β-D-fructosidase (FruA).

Ftf is an enzyme involved in the metabolism of sucrose to fructose characterized as an extracellular homopolymer, levan (fructan). Next, the enzyme FruA performs a hydrolysis reaction of the formed fructan to fructose. Fructosidase is encoded by the FruA gene, which is identified in many different species of streptococci and in fungi. The mechanism of action of both enzymes allows them to effectively inhibit the sucrose-dependent adhesion of the *S. mutans* pathway [63]. Authors have confirmed the inhibitory effect of FruA on the process of biofilm formation by *S. mutans*. To confirm this, a fructanase mixture (the mixture used was derived from a culture of *Aspergillus niger* and showed significant FruA activity) containing exo- and endo-inulinase was added for the cultivation of this bacterium. It was shown that these enzymes did not affect bacterial growth inhibition; however, they strongly inhibited their ability to form biofilm [63]. In addition, the ability of FruA fructosidase to use sucrose from the oral environment causes a lack of availability of this carbohydrate for *S. mutans*. This phenomenon is observed because FruA digests sucrose before *S. mutans* glucosyltransferases manage to produce the glucans necessary for biofilm formation [63].

Another microorganism strongly affecting *S. mutans*-induced biofilm in the oral cavity is *Lactobacillus* spp. (LB). LB bacteria are commensal microorganisms colonizing, among others, the human oral cavity. LB is strongly associated with the development of dental caries in the dentine, because it ferments sugars into acidic products, which decrease the pH in the oral cavity, and promote the development of biofilm (Fig. 3) [65]. On the other hand, low pH and antibacterial agents, such as hydrogen peroxide or bacteriocins produced by LB microorganisms, favor the purification of the oral cavity from microorganisms which are non-adaptive to such environmental conditions, for instance, *Porphyromonas gingivalis* [65]. Thanks to such properties, LB bacteria can be widely used as probiotics [34, 65–68]. LB microorganisms, by interacting with other microorganisms and the production of antimicrobial proteins, contribute to maintaining the balance in the natural flora of many ecosystems, e.g., the oral cavity [66].

Research conducted on various *Lactobacillus* microorganisms has confirmed the inhibitory effect of many of them on cariogenic microorganisms colonizing the oral cavity, among others *S. mutans*. Among the most active microorganisms are: *L. paracasei*, *L. plantarum*, *L. salivarius*, and *L. rhamnosus* [66]. It is also known that the above-mentioned probiotics do not affect the adhesive ability of *S. mutans*; however, they are able to inhibit the proliferation of the pathogen, and, thus, retard biofilm formation and development [68, 69]. Unfortunately, the mechanism of interaction between *Lactobacillus* and *S. mutans* has not yet been sufficiently investigated. Detailed in vitro studies focused on *Lactobacillus reuteri* [34, 65] have shown that different strains of this species interact with *S. mutans* with different forces. These interactions are dependent on the pH of the environment and the ability of probiotics to produce hydrogen peroxide and reuterin, an antibacterial protein formed from glycerol, resistant to proteolytic and lipolytic enzymes [24, 68]. The strains also differed in terms of their ability to adhere to surfaces coated with saliva. This feature is very important because bacteria unconjugated to the solid phase are quickly removed by swallowing [63]. Similar differences in activity against *S. mutans* have been observed among different strains of *Lactobacillus salivarius* [66].

The role of *Lactobacillus* bacteria to sustain oral purity is also a result of their specific coaggregation with *S. mutans* cells. This relationship applies to *L. paracasei* and *L. rhamnosus* [67]. The fusion of microorganisms in common aggregates prevents the adhesion of *S. mutans* to the surface of teeth and gums. This allows the removal of the pathogen from the oral cavity with saliva before the first stage of biofilm formation begins, that is, adhesion [67].

Tahmourespour et al. showed that a biosurfactant derivative produced by probiotic bacterium, *Lactobacillus fermentum*, inhibits biofilm formation. In the presence of *S. mutans*, the above-mentioned ability to form a derivative was inhibited, probably due to the inhibition of two key enzymes, GtfB and GtfC. A decrease in the gene expression encoding the mentioned glucosyltransferases in the presence of a biosurfactant derivative produced by *L. fermentum* [70, 71] has been reported. Ogawa et al. noted that *S. salivarius* can inhibit biofilm formation. Exo-beta-D-fructosidase synthesized by *S. salivarius* blocks the synthesis of polysaccharides, which reduces the amount of extracellular matrix produced by *S. mutans* [63].

Interactions occurring between *S. mutans* and other microorganisms in the oral cavity may contribute to both the development as well as the inhibition of biofilm formation. Through ongoing research, we have obtained new data on these interactions which enables the search for and use of novel, more specific and effective measures for the prevention of and fight against dental caries. In this fight, it is important to utilize, apart from synthetic products, the potential of natural products, such as probiotics and specific antimicrobial proteins. These substances exhibit a significantly lower number of side effects than artificially made specimens and are suitable for use under specific environmental conditions, which allows a high level of activity of the specimens to be maintained over a long period of time.

## Inhibition of biofilm formation—treatment perspectives in dental caries

### First, second, and third-generation medicines (phenolic compounds, chlorhexidine, delmopinol)

The growing problem of strain resistance to antibiotics compels us to seek other methods which deal with pathogens that occur in the oral cavity. Nowadays, we know of a number of substances with effective antimicrobial activity inhibiting biofilm development, for instance, chlorhexidine [72], delmopinol [73], or phenolic compounds [74]. Unfortunately, most of these substances cause side effects such as vomiting, diarrhea, addiction, or teeth discoloration. Therefore, alternative substances of antibacterial activity are being sought which would be safe for the users [33, 75].

### “Liquid enamel”—calcium phosphate

For many years, the primary role in the fight against dental caries has been played by calcium phosphate, which possesses remineralization properties [76–78]. A special calcium phosphate resin, which was designed for the gradual release of large amounts of these elements in places that require reconstruction, was established very quickly. Later, this compound gained the form of a nanoparticle of amorphous calcium phosphate composite (NACP), which was also as widely used in dentistry as its precursor. The advantages of the nanocomposite are:

- Improved mechanical features for its use in dentistry as compared to the classic combination of elements;
- Increased release of ions in acidic environments favoring the formation of voids and dental caries;
- Fast neutralization and raising of low pH to a safe level (about 6).

[76, 77].

### Nanoparticles of quaternary ammonium salts (QAS)

In the search for a substance which, apart from remineralization properties, would possess antibacterial ability, NACP was enriched with quaternary ammonium salts (QAS). A specific application was found for a quaternary ammonium called dimethacrylate (QADM). This compound has two active antibacterial domains at its ends, which increase the desired properties, and, additionally, mixes with other dental media easily [77]. Thanks to this change, a new composite of strong antibacterial activity was found. Its application results in shortening microorganism viability both as a planktonic form and in biofilms among others (*Streptococcus* sp. and *Lactobacillus* sp.), decreasing acid production by bacteria and a reduction of microorganism metabolic activity [76, 77]. This is possible thanks to the amphiphilicity of QAS compounds, which allow for these substances to react with the lipid part of the cell membrane and interfere with its function. As a result, it indirectly affects the activity of enzymes involved in the transport of substances through the membrane lipid, and, thus, alters the metabolic activity of bacterial cells [79].

### Antibacterial nanoemulsions

Lethal QAS properties against a broad spectrum of microorganisms have also been used in antibacterial nanoemulsions. In this context, cetylpyridinium chloride (CPC) was used [80]. Antibacterial nanoemulsion is characterized as a dispersing substance, namely, water and a lipid substance composed of surfactant, which forms nanoemulsion droplets. It is not toxic to humans or animals; however, it exhibits antibacterial, antifungal, and antiviral activity. Antibacterial properties of the emulsion result from the activity of nanodrops on bacterial cell membranes destabilizing the integration of its lipids [80].

### Cetylpyridinium chloride (CPC)

CPC has the ability to inhibit Ftfs enzymes (fructosyltransferases), which play an important role in microorganism-induced biofilm formation in the oral cavity. Thanks to this property, CPC plays the role of an antibacterial substance (affecting the cell membrane of a microorganism) and a substance inhibiting the development of biofilm. Increasing the efficiency of nanoemulsions through CPC enrichment has led to their wider application in products for oral hygiene, such as toothpastes and mouthwashes, and in dental materials, e.g., varnishes and dental fillings [80].

### 12-methacryloyloxydodecylpyridinium bromide (MDPB)

Among QAS, 12-methacryloyloxydodecylpyridinium bromide (MDPB) has also found an application in the fight against dental caries [81, 82]. The properties of MDPB and the possibility of its application have attracted the attention of research groups [82]. In experiments, the authors have investigated the effect of MDPB on the bacterial flora of the oral cavity, interactions with dental materials, and the possibility of a synergistic effect of this compound with nanoparticles of silver (NAg).

MDPB is a monomer characterized by antibacterial activity against aerobic and anaerobic bacteria isolated from translucent zones (i.e., *Actinomyces*, *S. mutans*) and antifungal activity (e.g., *Candida albicans*) [82]. Stiffened by polymerization of the filling bonding layer, it remains active against pathogens and, at the same time, does not negatively affect either human cells or the binding capacity of the filling material [82].

### Silver nanoparticles

Silver compounds became the research objective for Cheng et al. [81], who drew attention to their application in conjunction with QADM. Silver is known for its lethal activity against a wide spectrum of bacteria, viruses, and fungi. Its antibacterial activity is due to the ability of cell membrane disintegration, internal penetration, and destruction of intracellular organelles. An additional mechanism allowing for the inactivation of bacterial enzymes by inhibiting bacterial DNA replication capability enhances the efficacy of silver compounds. Low toxicity and long-term antibacterial activity due to the gradual release and lower resistance of bacteria against these compounds in relation to antibiotics are other advantages in the fight against microorganisms [81–83]. The sheer number of mechanisms and properties describing the activity of substances containing silver particles lead to the inability for microorganisms to sustain full protection against their effects. An additional difficulty is the role of silver compounds as catalysts, rather than substances participating in chemical reactions [84].

Silver nanoparticles have a huge active surface, and, therefore, their level in dental composites is estimated at only 0.05 to 0.1 % by weight. This proportion provides both effective antibacterial activity as well as a lack of effect on the mechanical filling properties [81].

### NAg with MDPB and NAg with QADM

Research conducted by both groups showed that the combinations of NAg with MDPB and NAg with QADM give much better results than the use of these substances alone. Based on Table 3, it can be observed that the combination of silver nanoparticles with MDPB and QADM does not affect the mechanical properties of the dental material and is not toxic to human cells. A major advantage of such combinations is the increase in the ability to inhibit microorganism growth, as well as the significant reduction in their metabolic activity and vitality.

**Table 3:** The effect of MDPB + NAg and QADM + NAg on chosen traits (the table was prepared based on data published by Cheng et al. [81] and Zhang et al. [82])

Trait	Control	MDPB + NAg	QADM + NAg
Growth inhibition zone	1 mm	10-fold higher	8-fold higher
The strength of binding of dental material	30–32 MPa	No changes	No changes
Metabolic activity of bacteria measured by absorbance in the enzymatic assay (reduction of MTT^a^ at 540 nm wavelength).	0.5 A_540_/cm^2^	0.05 A_540_/cm^2^	No changes
Viability of bacteria described as a ratio of the number of live cells to dead cells based on the image from a fluorescence microscope	Long lifetime	Significantly lowered in comparison to control	Significantly lowered in comparison to control
CFU (colony forming unit, number of microorganisms in the study material) after application of antibacterial agents	23 × 10^6^ for control MDPB 2.5 × 10^6^ for control QADM	0.5 × 10^6^	0.9 × 10^4^

^a^MTT – tetrazolium dye

### Variations of silver nanoparticles with MDPB, QADM, and with silver diamine fluoride (SDF)

The combination of silver with fluoride led to the formation of silver diamine fluoride (SDF), characterized by antibacterial properties, for which the metal component is responsible, and remineralization properties due to the presence of fluoride [85]. It has been shown that SDF can be used as an element of dental caries prevention [86], as well as a substance inhibiting the development of disease by reducing the amount of cariogenic microorganisms in the oral cavity and that it has a positive influence in zones of tooth enamel demineralization [85]. During studies on the described compound, the sensitivity of *S. mutans* and *Actinomyces naeslundii* to its function was also demonstrated [87].

However, studies examining the effects of SDF on dual-species biofilm formed by *S. mutans* and *Lactobacillus acidophilus* illustrate much better the effectiveness of this compound in the natural oral environment, in which biofilm is characterized as a structure of multi- and not single species. Mei et al. [72] showed that, in dual-species biofilm, the antibacterial activity of silver ions is much lower than in the biofilm of single species; however, it does not disappear completely. This is confirmed by the ratio of dead and live cells, which, for the control, was equal to 0.02, whereas for the samples with SDF addition, it was 6.74. These studies, apart from confirming SDF antibacterial activity against *S. mutans* and *L. acidophilus*, also proved that this substance slows the process of enamel demineralization and protects collagen from damage. This dual action of SDF may contribute to a wider use in oral hygiene products and to clinical success in the fight against dental caries.

### Natural inhibitors: chitosan

Antibacterial substances which could be included in the formulations of oral hygiene products and would not exert side effects are still being researched. One such substance is chitosan, a polysaccharide formed by the N-acetylation reaction of chitin [88]. Both compounds are present in the plant, fungi, and animal worlds, and have natural antibacterial and antifungal properties. A limitation in the use of chitosan for oral hygiene products, however, was proven by its insolubility in water, because the compound is dissolved only in acids. Attempts have been made to modify this property, and as a result of the Maillard reaction or sugar modification, water-soluble chitosan of unchanged antibacterial properties was obtained. It has been shown [88] that such modified chitosan exhibits the highest antibacterial activity against microorganisms isolated from the oral cavity in an environment with a pH range between 5 (for *Klebsiella pneumoniae*, *Streptococcus mutans*, *Staphylococcus aureus*) and 8 (for *Enterobacter gergoviae*, *Lactobacillus brevis*, *Staphylococcus saprophyticus*).

The optimum temperature for chitosan activity, in which the antibacterial activity remained at the level of 50–96 %, was estimated at 37 °C in relation to the above-mentioned bacterial species. For *K. pneumoniae*, *L. brevis*, and *S. saprophyticus*, the minimum bactericidal concentration of chitosan (MBC) was 500 μg/ml, whereas for other species, it was 400 μg/ml. It was also shown that 5 s is sufficient for chitosan to exhibit antibacterial activity in relation to the above-mentioned microorganisms species at the level of 99.6 %, and 20s was required to reach the level of 99.9 %.

Thus, it was proven that water-soluble chitosan has a comparable efficacy in the fight against dental caries in relation to commonly used antibacterial mouthwashes for oral hygiene. A very strong argument for the use of chitosan in such formulations is also its much lower toxicity than the conventionally used alcohols, chlorhexidines, or cetylpyridinium chloride [88], which are commonly used in products for oral cavity disinfection in diseases of the upper respiratory tract, e.g., Tantum Verde.

### Natural inhibitors—antimicrobial peptides (AMPs): chrisofsin-1, D-Nal-Pac-525

Another safe and potential solution to the problem of dental caries in humans are antimicrobial peptides (AMPs). They exhibit a lethal effect against many microorganisms but do not cause side effects for humans [33].

AMPs represent a family of short polypeptides, typically associated with congenital human and other organism immune systems. An example of such a protein is chrisofsin-1 amphipathic, an α-helical protein of broad activity spectrum against both Gram-negative and Gram-positive bacteria. Chrisofsin-1 possesses a hydrophilic domain at the C-terminal end of the polypeptide chain.

Via electrostatic interactions with negatively-charged phospholipids of bacterial cell membranes, it covers the membrane tightly, and then the hydrophilic domain penetrates into the structure of the membrane, forming a number of pores inside. Wang et al. observed the first changes in cell shape during their studies [33] using chrisofsin-1 at concentrations of 2–4 mg/ml (depending on the bacteria species). Thus, chrisofsin-1 exhibits potent destructive action against lipid components of microorganism cell membranes, such as *S. mutans*, *S. sanguinis*, *S. sobrinus*, *S. gordonii*, *Actinomyces* sp., and *Lactobacillus* sp. and, thus, it is able to induce disintegration and bacterial lysis.

This contributes to reduced viability of pathogens, both as planktonic forms as well as those grouped in biofilm. Compared to *S. mutans*, chrisofsin-1 exhibits lethal activity after 30 min with the use of an 8 times higher concentration than the minimum inhibitory concentration (MIC). For comparison, *E. faecalis* was destroyed after 5 min and *L. fermentis* after 60 min with the use of 12 times the MIC. Among the strains under investigation, only *A. viscosus* was observed to be more resistant than *S. mutans*. Its eradication required the application of 8 times the MIC for 60 min. This shows the different effects of chrisofsin-1 in relation to particular bacterial species [32].

Microorganisms remaining in the biofilm structure are 10–1,000 times more resistant than those in the planktonic phase. For this reason, AMPs could be used most effectively in the early stages of biofilm formation. It would prevent its growth and the colonization of the oral cavity by other pathogens. In the phase of biofilm maturation, AMPs would only delay the cariogenic process [33].

The D-Nal-Pac-525 peptide appeared to be another example of an AMP with high antibacterial activity. The high anticaries activity of D-Nal-Pac-525 peptide is associated with the tryptophan conversion in the tryptophan-rich (Trp-rich) Pac-525 peptide fragment to the D-β-naphthylalanines fragment [62]. Studies with the use of scanning and transmission electron microscopes have shown that the D-Nal-Pac-525 peptide causes morphological changes and cell membrane damages of *S. mutans* with a MIC of 4 ug/ml, while the inhibition of biofilm formation was observed under an MIC of 2 ug/ml. The effective anti-biofilm protection of the D-Nal-Pac-525 peptide was highlighted in the first stage of its formation (planktonic and colonization phase of bacteria) (Fig. 2), with no effect on pre-existing bacterial biofilms. The above-mentioned results are comparable to beta-defensin (6 mM) action in human saliva. Data suggest the use of AMPs exemplified by D-Nal-Pac-525 as new therapeutic agents which combat the problems caused by the occurrence of bacterial strains resistant to antibiotics [62].

The problem of infectious diseases is inherently associated with the growing number of microorganisms becoming resistant to a broader spectrum of antibiotics (multidrug resistance). Therefore, the attention of scientists has increasingly been drawn to antibacterial, antifungal, and antiviral substances of natural origin. Many metabolites of plant origin, such as polyphenols, alkaloids, tannin, terpenoids, steroids, or flavonoids, are known for such properties [61, 89, 90]. Because of their proven antibacterial activity, these compounds act as supplements in oral hygiene products.

Most of the above-mentioned substances have found an application in dentistry. They are included in composites and dental restorative resins, the properties of which allow for the reduction of biofilm formation and reduce the probability of dental caries episodes at the same place. They have been introduced into treatment because commonly used methods of treating developing dental caries usually do not allow for the removal of bacteria from the mineralized tooth tissues. In such situations, it is suggested that antibacterial agents should be applied on already developed dentin [91]. These are usually fillings which release ions [92] in the form of dicalcium phosphate anhydrous nanoparticles (DPCA) or tetracalcium phosphate (TTCP) often enriched with fluorine [92], or modifications of such fillings, e.g., with the addition of QAS or silver nanoparticles [76, 77, 81, 82].

### Iodine compounds

Studies on substances allowing for the reduction of dental caries incidence also include iodine compounds such as potassium iodide or povidone–iodine (PVP-I). PVP-I is a complex of iodide and polyvinylpyrrolidone, which is a specific “reservoir” of free iodine ions forming an active domain of the whole structure. Iodine slowly released from the complex is able to penetrate the bacterial membrane and enter into the cytosol, where it deactivates the key proteins, fatty acids, and nucleotides in the metabolism of the cell [93]. Additionally, the slow release of iodine reduces its toxic effects for human cells [94] and its short-term use does not irritate even damaged oral mucosa. No side effects, such as discoloration of teeth, tongue, or taste change, have been reported [95].

Hosaka et al. [95] decided to test the antibacterial activity of PVP-I in relation to the two microorganisms associated with oral cavity diseases, *Porphyromonas gingivalis* and *Fusobacterium nucleatum*. They showed that PVP-I, at a concentration of 7 %, is able to reach the activity allowing 100 % of *P. gingivalis* species to die after 3 min, and an identical effectiveness against *F. nucleatum* with the use of 5 % of PVP-I was observed after 30 s. The results obtained for dual-species biofilm formed by the microorganisms under investigation indicated that this biofilm is 200 times more resistant to the activity of the tested substance than mono-species cultures, although the activity of 5 % PVP-I allows bacteria which form this biofilm to be killed.

Unfortunately, it has also been shown that the concentrations of PVP-I (0.23–0.47 %) used in common mouthwashes are not sufficient for a significant reduction of *P. gingivalis* and *F. nucleatum* biofilm formation ability during a time of 30 s to 1 min, which was estimated as the average time of mouthwashing by the public [95]. Based on these reports, one may conclude that other bacterial species will also exhibit a different sensitivity to PVP-I activity, and a multi-species biofilm will be much more resistant to both planktonic cells as well as structures formed by a lower number of microorganisms.

The antibacterial activity of PVP-I has also been tested for *S. mutans* strains isolated from children with early childhood caries [96, 97]. It has been proven that the application of 10 % PVP-I on healthy teeth and on teeth with dental caries every 3 months for 1 year significantly reduces the number of these microorganisms in the oral cavity as compared to baseline levels [96, 98]. This reduced number of cariogenic bacteria is, therefore, likely to reduce the probability of childhood dental caries. Similar conclusions can be drawn from studies conducted on a group of 172 children, which showed that combination therapy of dental caries through the application of 10 % PVP-I and dental varnish containing 5 % sodium fluoride reduces the incidence of new carious lesions by 31 % in relation to the application of the varnish alone [86].

### Ursolic acid

Composite resins containing ursolic acid which inhibited the biofilm formation of *S. mutans* at a concentration of 0.1–0.5 wt.% [99] have been proposed as another solution to the problem of dental caries. This compound has similar properties to those tested in clinical trials: triterpenoids of recorded anticancer, antiwrinkle, and antibacterial activity.

### Aromatic amino acid pathways

There is little information concerning the effect of amino acids on the ability of *S. mutans* to form biofilms. Kolodkin-Gal et al. conducted a study on biofilm structure decomposition on the example of *Bacillus subtilis*. This microorganism produced a factor which inhibited biofilm formation and next induced structure disassembly. Analysis of this factor’s composition demonstrated that it is a mixture of four D-amino acids: leucine, methionine, tyrosine, and tryptophan [100]. A similar inhibitory effect of the D-amino acid mixture was observed in the case of biofilm formation by *Staphylococcus aureus* [101] and *Pseudomonas aeruginosa* [102]. Recent studies have indicated that increased levels of L-tryptophan itself may also give the signal for biofilm decomposition; a suitable experiment was conducted on the example of biofilm formed by *E. coli* [103]. Our study indicates that another aromatic L-amino acid, i.e., tyrosine, has similar properties and may inhibit biofilm formation by *S. mutans* (paper submitted to the editorial board). It needs to be verified as to whether other aromatic amino acids may exhibit anti-biofilm formation activity for *S. mutans* species, and also whether inhibition caused by these compounds is limited to ex vivo models, as well as whether and to what degree they may be applied in clinical situations (proposed animal model).

The increasingly broader range of substances protecting us from the occurrence of carious lesions while not causing side effects creates hope for the design of oral hygiene products allowing for the effective fight against this disease and for the complete protection of children from the development of early caries lesions in primary teeth.

## Summary

Studies on biofilm formed by *S. mutans* clearly show that the virulence of *S. mutans* strains is dependent on environmental conditions, and, thus, on in vivo model host-dependent characteristics. On the other hand, to form a biofilm structure, it is not sufficient to ensure appropriate environmental conditions. Microorganisms alone must possess characteristics which, in terms of the above-mentioned favorable conditions, will allow the adhesion and formation of microcolonies [6, 35, 36].

Cariogenic strains of *S. mutans* exhibit large variation due to the occupation and expression of characteristics considered as virulence determinants: (A) most *S. mutans* strains exhibit the ability to survive and reproduce in acidic pH, without eliminating the possibility to survive in alkaline medium levels (described in case studies isolated cases of bacteremia), (B) most *S. mutans* strains possess the activity of specific proteins (enzymes) associated with biofilm formation, depending on a number of biochemical properties of the host (EPS production, the condition of the host immune system).

Biofilm formation in the oral cavity is a complex process, dependent not only on EPS produced by *S. mutans* and the host, but also on other species colonizing the oral cavity. The occurrence of potentially non-pathogenic species in the oral cavity environment seems to be the key point.

This study is an introduction to the research which aims to confirm or exclude the virulence of selected *Streptococcus* strains in clinical material, as well as shedding new light on the development of potential compounds blocking the metabolism of *Streptococcus mutans* through influence on specific proteins involved in the formation of the complex structure which is a biofilm.
